# Development and validation of an interpretable machine learning model for predicting enteral nutrition-associated diarrhea in ICU patients: a multicenter study

**DOI:** 10.3389/fnut.2026.1896922

**Published:** 2026-08-17

**Authors:** Yuhong Wang, Wei Hu, Caiyue Xu, Xinwei Zhang, Fengzhi Chai, Yunfan Ji, Di Xu, Xia Li

**Affiliations:** 1 School of Nursing, Jinzhou Medical University, Jinzhou, Liaoning, China; 2 Department of Nursing, Shangrao People’s Hospital, Shangrao, Jiangxi, China; 3 First Affiliated Hospital of Jinzhou Medical University, Jinzhou, Liaoning, China

**Keywords:** critically ill patients, enteral nutrition, enteral nutrition-associated diarrhea, machine learning, nursing, prediction model

## Abstract

**Background:**

ICU patients receiving EN are at high risk of ENAD, which may adversely affect clinical outcomes and increase mortality risk. Early identification of patients at high risk of ENAD may facilitate timely risk assessment and the implementation of individualized nutritional management. This study aimed to develop and externally validate an interpretable ML-based model for predicting ENAD risk in critically ill patients.

**Methods:**

This retrospective multicenter study included critically ill patients receiving EN at the First Affiliated Hospital of Jinzhou Medical University between January 2024 and December 2025. An independent external validation cohort was obtained from Shangrao People’s Hospital during the same period. Twelve ML models were developed using R software. Model performance was evaluated using AUC, accuracy, precision, NPV, recall, and F1 score. Calibration curves and DCA were used to assess calibration and clinical utility. SHAP analysis was performed to visualize feature importance.

**Results:**

Among the 12 models, RF showed the best discriminative performance, with an AUC of 0.811 (95% CI: 0.769–0.853) in the test set and 0.808 (95% CI: 0.759–0.856) in the external validation cohort.

**Conclusion:**

The externally validated random forest model showed acceptable discriminative performance and provided an interpretable assessment of the contributions of individual features. The model may support static early risk stratification of ENAD among critically ill patients receiving enteral nutrition. However, the available evidence remains insufficient to support its routine clinical implementation. Further validation in prospective studies, independent populations, and longitudinal clinical datasets is required.

## Introduction

Patients admitted to intensive care units (ICUs) often present with severe illness, systemic inflammatory responses, hypercatabolism, impaired nutrient utilization, and varying degrees of multiple organ dysfunction (1). Owing to impaired consciousness, mechanical ventilation, and gastrointestinal dysfunction, many ICU patients are unable to achieve adequate oral nutritional intake (2). In addition, prolonged physiological stress and negative nitrogen balance may further increase the risk of malnutrition, which can impair immune function, delay tissue repair, and potentially contribute to disease progression, complications, and short-term mortality (3). Therefore, nutritional support is an essential component of comprehensive ICU management.

Enteral nutrition (EN) is generally considered the preferred route of nutritional support for eligible ICU patients. Compared with parenteral nutrition, EN is more consistent with physiological metabolic processes and may help preserve gastrointestinal mucosal integrity, support intestinal barrier function, and contribute to meeting patients’ metabolic requirements (4). According to clinical guidelines issued by the Society of Critical Care Medicine and the American Society for Parenteral and Enteral Nutrition (ASPEN), EN support should be initiated as early as possible, preferably within 24–48 h after ICU admission, in patients without clear contraindications who are unable to meet their nutritional requirements orally (5). Despite these potential benefits, enteral nutrition-associated diarrhea (ENAD) remains a common and clinically important complication in ICU practice (6, 7). Previous studies have reported wide variation in the incidence of diarrhea among critically ill patients receiving EN, with ENAD occurring in approximately 20%–30% of patients in some cohorts (8). ENAD may interrupt nutritional support, compromise the achievement of nutritional targets, increase nursing workload and medical costs, and prolong hospitalization. It has also been associated with an increased risk of complications and short-term mortality.

Enteral nutrition-associated diarrhea is generally considered to result from the combined effects of multiple interacting factors rather than from a single cause (9). Patient-related factors, including advanced age, disease severity, gastrointestinal disease, and hypoproteinemia, may reduce tolerance to EN and increase diarrhea risk (10, 11). Formula composition may influence gastric digestion and gastrointestinal tolerance (12, 13). Drug exposure, particularly broad-spectrum antibiotics, proton pump inhibitors, and prokinetic agents, may also alter normal gastrointestinal function (14, 15). Nursing-related factors, including the storage, handling, and warming of enteral formulas, may also influence feeding tolerance or the risk of formula contamination (16). These complex and interacting factors highlight the need for accurate risk assessment tools to identify patients at high risk of ENAD.

Traditional clinical prediction models, such as logistic regression, are widely used because of their transparency and interpretability. However, in conventional implementations, logistic regression often requires prespecified functional forms and interaction terms, which may limit its ability to capture complex non-linear associations and high-order interactions in multidimensional clinical data (17, 18). In recent years, by integrating multidimensional clinical information and accommodating non-linear associations and complex interactions in high-dimensional data, machine learning may provide a useful complement to traditional statistical methods for ENAD risk prediction (19, 20). Although machine learning methods have increasingly been applied to clinical risk prediction, studies specifically addressing ENAD risk prediction remain relatively limited. Previous studies have generally been based on single-center cohorts or relatively specific clinical populations, and the generalizability of their findings to other ICU settings requires further evaluation (21). In addition, existing models have primarily focused on demographic characteristics, comorbidities, and indicators of disease severity, with comparatively limited consideration of enteral nutrition practices, medication exposure, nursing-related variables, and other factors associated with clinical management (22). The lack of independent external validation and insufficient reporting of the temporal definitions of predictors may also limit model reproducibility and clinical interpretability to some extent (23, 24). Moreover, the interpretability of some machine learning models remains limited, which may affect clinicians’ understanding of model outputs and their subsequent clinical application (25, 26).

Against this background, the present study aimed to develop and externally validate an ENAD risk stratification model with interpretable outputs using routinely available ICU data. Candidate predictors encompassed multiple domains, including demographic characteristics, clinical conditions, treatment-related factors, enteral nutrition practices, nursing-related variables, and laboratory findings. Several commonly used machine learning algorithms were compared, and SHapley Additive exPlanations (SHAP) were applied to assess the relative contributions of individual predictors to model outputs (26). This analysis was intended to characterize variables with relatively greater contributions to the model predictions, including those related to nutritional support and nursing care, without implying causal relationships.

## Materials and methods

### Study population

**FIGURE 1:**
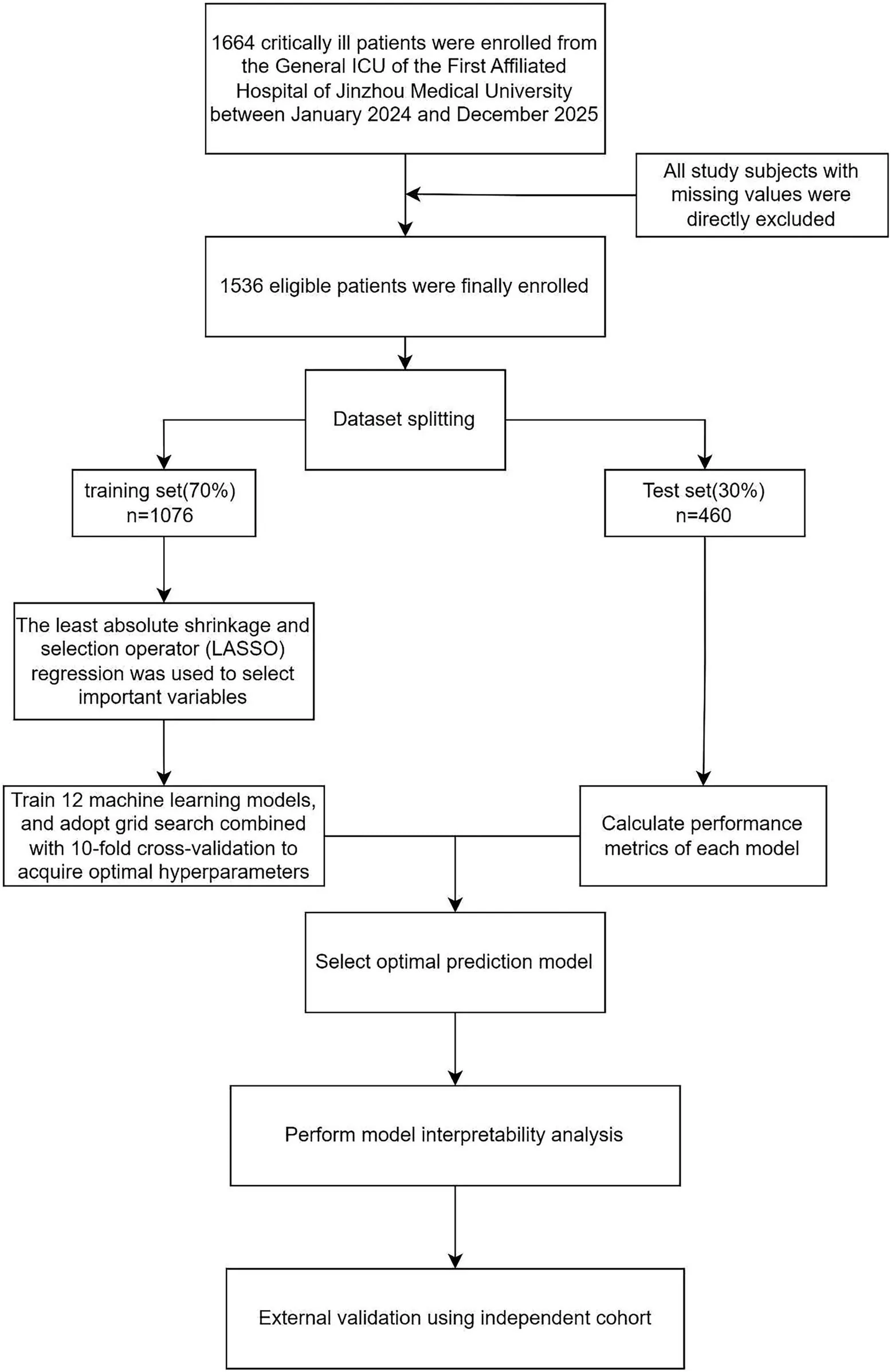
Study flowchart of the prediction model for enteral nutrition-associated **diarrhea** in **intensive care unit** (ICU) patients.

This study was designed as a dual-center retrospective cohort study. Adult critically ill patients who were admitted to the intensive care unit (ICU) of the First Affiliated Hospital of Jinzhou Medical University between January 2024 and December 2025 and received EN support were consecutively screened. This cohort was used for model development and internal validation. After applying the prespecified inclusion and exclusion criteria, a total of 1,536 patients were included in the development cohort. In addition, 356 eligible patients admitted to Shangrao People’s Hospital during the same period were included using the same eligibility criteria and served as an independent external validation cohort to assess the generalizability of the model. The overall study workflow is shown in Figure 1.

The sample size was evaluated according to the modified events-per-variable (EPV) criterion proposed by Vabalas et al. (27), which recommends an EPV of at least 15 for non-linear models. In the development cohort, 534 outcome events were observed, and 42 candidate predictors were included, yielding an actual EPV of 12.71, which was slightly below the recommended threshold. To reduce the risk of model overfitting, regularization techniques and 10-fold cross-validation were applied during model development, and model generalizability was further evaluated using an independent external validation cohort.

The inclusion criteria were as follows: age ≥ 18 years; ICU length of stay >48 h; receipt of EN therapy for >48 h; and initiation and implementation of EN in accordance with the ESPEN guideline recommendations regarding the indications (23, 24), timing, and contraindications of EN in critically ill patients (28). The exclusion criteria were as follows: diarrhea at ICU admission, a history of intestinal disease or gastrointestinal surgery within the previous 30 days, expected survival time < 24 h, or concurrent participation in other nutrition-related interventional trials. This study was approved by the Ethics Committee of Jinzhou Medical University (JZMULL2025270) and the Ethics Committee of Shangrao People’s Hospital (2024311).

### Data collection

Patient data were retrospectively extracted from the hospital electronic medical record system and ICU nursing records. A standardized database was established using EpiData, and data extraction was independently performed by two trained researchers. Variable definitions, entry criteria, and coding rules were predefined. Missing, abnormal, or inconsistent data were verified against the original medical records and treated as missing if they could not be confirmed.

Based on previous literature, the proposed mechanisms and clinical relevance of ENAD, data availability, and multidisciplinary expert input, 42 candidate predictors were selected. To minimize the risk of data leakage, all predictors were restricted to information available before ENAD onset or within the predefined predictor ascertainment window. This window extended from ICU admission to 48 h after the initiation of EN, with reference to guideline recommendations that EN be initiated within 24–48 h in critically ill patients. This definition was intended to establish an early risk assessment setting and avoid the inclusion of information recorded after ENAD onset. For patients who developed ENAD, only laboratory, nursing, medication, treatment, and EN-related variables recorded before the first ENAD episode were included. For patients who did not develop ENAD, candidate predictors were restricted to information available within 48 h after EN initiation. The same variable extraction rules and temporal definitions were applied to the training set, internal test set, and external validation cohort to reduce temporal heterogeneity and information bias and to improve comparability across cohorts.

The 42 candidate predictors were grouped into five domains: demographics, clinical and environmental factors, treatment-related interventions, EN-related variables, and laboratory indicators. These included sex, age, ICU admission source, marital status, comorbidities, ICU admission diagnosis, Glasgow Coma Scale (GCS) score, Acute Physiology and Chronic Health Evaluation II (APACHE II) score, ICU stay, mechanical ventilation duration, ICU room temperature, Nutritional Risk Screening 2002 (NRS 2002) score, hematochezia, ICU ward type, renal replacement therapy, antibiotic-related variables, gastrointestinal medications, sedatives, opioids, oral potassium preparations, probiotics, EN formula type, feeding mode, catheter type, EN duration, and laboratory parameters including WBC, Hb, Alb, BUN, serum sodium, serum potassium, CRP, and PCT. Laboratory values were defined as the measurements closest to EN initiation before ENAD onset or within the predefined prediction window.

### Data processing

All analyses were performed using R software version 4.5.1, and samples with missing values were excluded. Non-normally distributed continuous variables were expressed as medians with interquartile ranges and compared using the Mann–Whitney U test. Categorical variables were summarized as frequencies and percentages and compared using the chi-square test. A two-sided *p*-value < 0.05 was considered statistically significant, with exact *p*-values reported except when *p* < 0.001. The development cohort was randomly divided into a training set (*n* = 1,076) and an internal test set (*n* = 460) at a ratio of 70:30 using stratified random sampling based on whether ENAD occurred. Sampling was performed separately among patients with and without ENAD to maintain approximately the same proportion of ENAD events in both subsets as in the original development cohort. Feature selection and model development were conducted exclusively in the training set, whereas the internal test set was retained as an independent dataset for model evaluation to minimize data leakage and ensure reliable performance assessment.

### Definition of diarrhea

Enteral nutrition-associated diarrhea was defined according to previously published criteria (22), requiring the concurrent presence of abnormal stool consistency, classified as Type ≥ 6 on the Bristol Stool Form Scale (29), and increased stool frequency and/or volume, defined as ≥3 bowel movements per day or total stool output exceeding 500 g/24 h.

### Statistical analysis and model development

Feature selection was performed in the training set using least absolute shrinkage and selection operator (LASSO) regression with 10-fold cross-validation, with λ set to lambda.1se. The test set was kept completely isolated throughout this process to avoid data leakage. The selected features were subsequently used to develop 12 machine learning models, including logistic regression, random forest (RF), decision tree, support vector machine, XGBoost, AdaBoost, K-nearest neighbors, LightGBM, neural network, naïve Bayes, artificial neural network, and multilayer perceptron. Notably, although neural networks, artificial neural networks, and multilayer perceptrons all fall within the broader family of neural network methods, they were implemented in this study using three independent coding pipelines. Each model was independently programmed, trained, and tuned, with its own model construction and hyperparameter optimization procedures. Accordingly, the three models were evaluated as distinct implementations rather than as fundamentally different algorithmic categories in terms of their underlying learning principles. Their separate inclusion was intended to compare the predictive performance of independently constructed neural network architectures within a consistent data-partitioning and evaluation framework. Hyperparameters for each model were optimized using grid search combined with 10-fold cross-validation, as detailed in Supplementary File 1. Model performance was evaluated in the test set using the area under the receiver operating characteristic curve (AUC), accuracy, precision, recall, F1 score, and negative predictive value, and the best-performing model was selected. Calibration curves, decision curve analysis (DCA), and Shapley additive explanations were further used to assess model calibration, clinical utility, and feature contributions, respectively. External validation was performed to evaluate the generalizability and stability of the final model.

## Results

### Baseline characteristics

A total of 1,536 ICU patients receiving enteral nutrition were included in this study, of whom 534 developed ENAD, corresponding to an incidence of 34.8%. Baseline characteristics of patients with and without ENAD are summarized in Supplementary Table 1. Compared with the non-ENAD group, the ENAD group had a higher proportion of transferred patients (*P* < 0.001). Regarding nutrition- and treatment-related factors, the ENAD group had higher proportions of patients with positive Nutritional Risk Screening 2002 results and positive fecal occult blood tests. The proportions of patients receiving high-energy whole-protein enteral nutrition formula (TP-HE) and mixed feeding were also higher in the ENAD group (all *p* < 0.05).

In addition, antibiotic use, intravenous antibiotic administration, and combined antibiotic therapy were more frequent in the ENAD group. The use of prokinetic agents, oral potassium supplementation, probiotics, and sedatives was also more frequent in the ENAD group than in the non-ENAD group (all *p* < 0.001). Regarding disease severity, the ENAD group had higher APACHE II scores and lower GCS scores (both *p* < 0.01). Moreover, the duration of mechanical ventilation, ICU length of stay, duration of enteral nutrition, and duration of sedative use were longer in the ENAD group (all *p* < 0.001). Laboratory results showed that WBC and Hb levels were lower in the ENAD group, whereas serum sodium levels were higher (all *p* < 0.05). No significant between-group differences were observed for the remaining variables.

### Feature selection using LASSO regression

**FIGURE 2:**
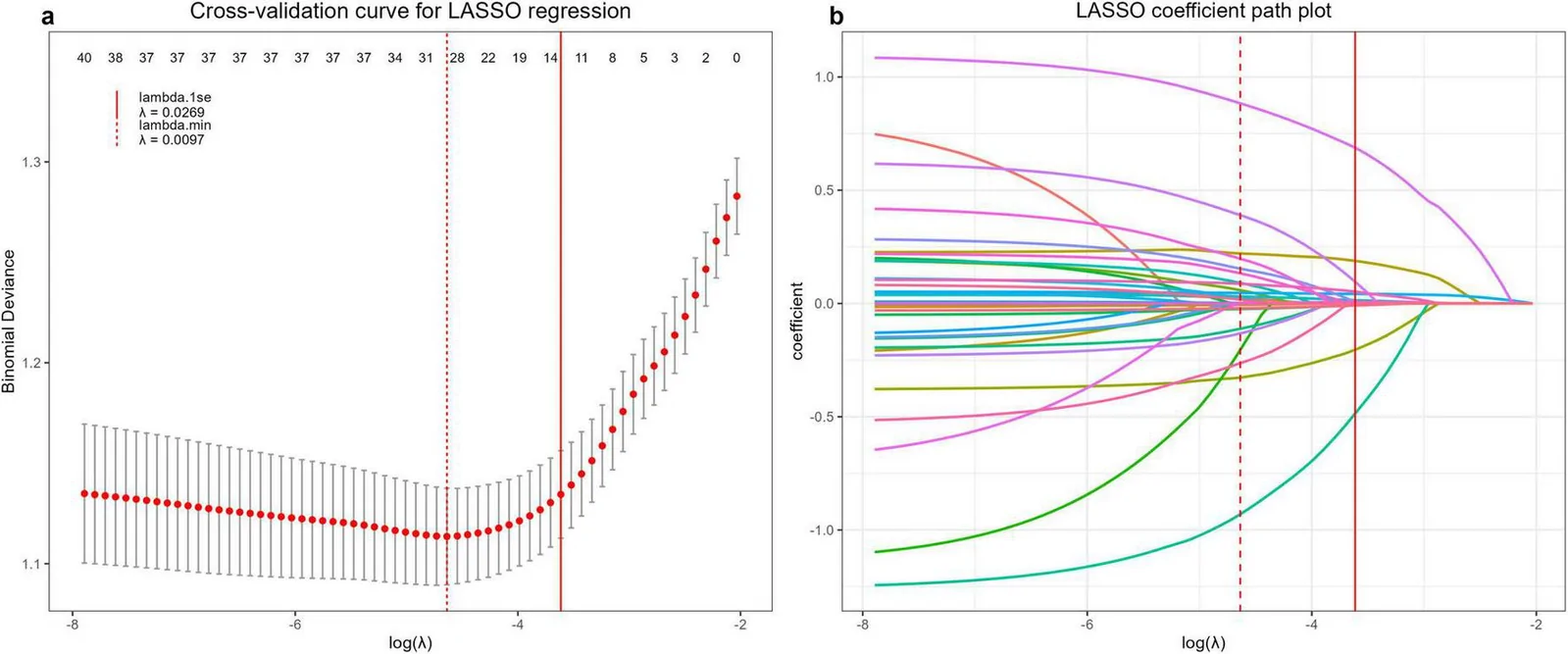
Results of predictor selection for enteral nutrition-associated diarrhea based on least absolute shrinkage and selection operator (LASSO) regression. **(a)** Cross-validation curve for LASSO regression. **(b)** LASSO coefficient path plot.

Based on the training set, LASSO regression with 10-fold cross-validation was used for feature selection to reduce multicollinearity and remove redundant predictors. λ = lambda.1se (0.026945) yielded a parsimonious and clinically interpretable feature set (Figure 2), identifying 12 key ENAD-related predictors: mechanical ventilation duration, room temperature, ICU length of stay, prokinetic agent use, sedation duration, GCS score, white blood cell count, probiotic use, combined antibiotic therapy, oral potassium use, H_2_-receptor antagonist use, and ICU unit type.

### Model development and performance evaluation

**FIGURE 3:**
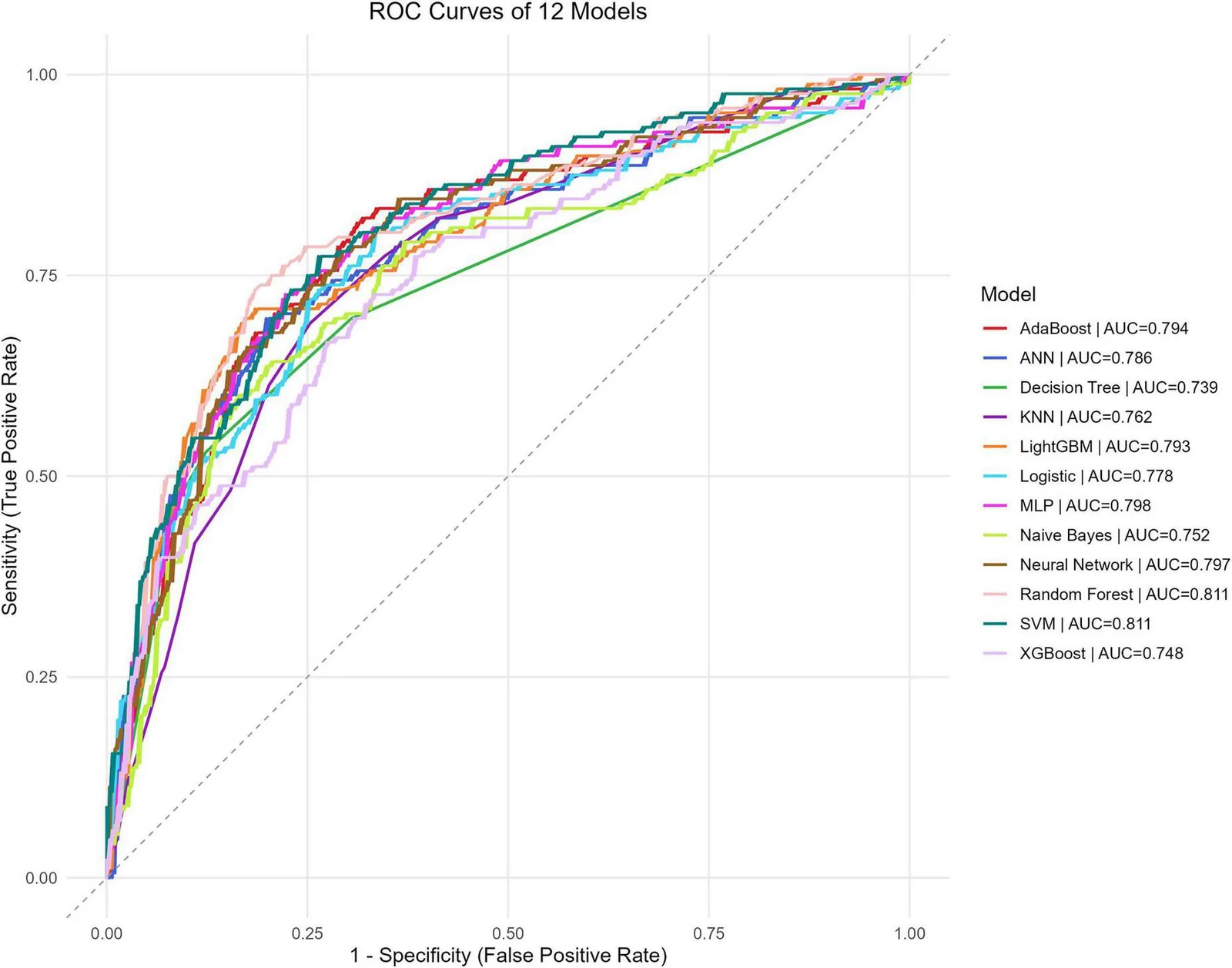
Receiver operating characteristic (ROC) curves of 12 machine learning models for predicting enteral nutrition-associated diarrhea in critically ill patients in the test set.

Based on the 12 selected features, 12 machine learning models were developed and evaluated in an independent test set. All models showed predictive ability, although their overall performance varied (Table 1). Among them, the RF model achieved the best overall performance, with an AUC of 0.811 (95% CI: 0.769–0.853), recall of 0.786 (95% CI: 0.722–0.824), F1 score of 0.710 (95% CI: 0.661–0.759), and negative predictive value of 0.860 (95% CI: 0.813–0.900) in the test set. The support vector machine (SVM) model also achieved a high AUC of 0.811, but its recall was lower than that of the RF model. LightGBM showed the highest accuracy (0.776), whereas the neural network model had the highest precision (0.724) but a relatively lower recall. The ROC curves further demonstrated that the RF model had superior overall discriminative performance compared with the other models (Figure 3); therefore, it was selected as the optimal prediction model in this study.

**TABLE 1:** Performance metrics (95% CI) of 12 machine learning models for predicting the risk of enteral nutrition-associated diarrhea in critically ill intensive care unit (ICU) patients.

Model	AUC (95% CI)	Recall (95% CI)	Accuracy (95% CI)	F1 score (95% CI)	Precision (95% CI)	NPV (95% CI)
Logistic	0.778 (0.732–0.824)	0.679 (0.606–0.750)	0.728 (0.687–0.765)	0.646 (0.589–0.699)	0.616 (0.549–0.677)	0.804 (0.753–0.849)
Random forest	0.811 (0.769–0.853)	0.786 (0.722–0.824)	0.765 (0.724–0.802)	0.710 (0.661–0.759)	0.647 (0.585–0.713)	0.860 (0.813–0.900)
decision tree	0.739 (0.692–0.785)	0.696 (0.629–0.767)	0.696 (0.654–0.737)	0.626 (0.569–0.677)	0.568 (0.502–0.636)	0.799 (0.752–0.849)
SVM	0.811 (0.770–0.853)	0.636 (0.567–0.707)	0.750 (0.711–0.791)	0.650 (0.593–0.705)	0.665 (0.595–0.733)	0.796 (0.749–0.839)
XGBoost	0.750 (0.703–0.797)	0.750 (0.682–0.812)	0.691 (0.648–0.730)	0.640 (0.580–0.691)	0.558 (0.491–0.620)	0.821 (0.768–0.869)
AdaBoost	0.794 (0.749–0.838)	0.738 (0.672–0.802)	0.739 (0.698–0.776)	0.674 (0.618–0.724)	0.620 (0.553–0.683)	0.831 (0.782–0.874)
KNN	0.762 (0.717–0.808)	0.690 (0.617–0.760)	0.726 (0.685–0.765)	0.648 (0.590–0.703)	0.611 (0.541–0.678)	0.807 (0.760–0.851)
LightGBM	0.793 (0.749–0.837)	0.708 (0.639–0.775)	0.776 (0.737–0.813)	0.698 (0.642–0.750)	0.688 (0.618–0.759)	0.829 (0.783–0.872)
Neural network	0.797 (0.753–0.837)	0.577 (0.506–0.649)	0.765 (0.730–0.804)	0.642 (0.579–0.699)	0.724 (0.648–0.794)	0.782 (0.739–0.825)
Naive bayes	0.752 (0.703–0.800)	0.702 (0.635–0.768)	0.693 (0.650–0.735)	0.626 (0.566–0.681)	0.565 (0.495–0.625)	0.801 (0.751–0.848)
ANN	0.786 (0.741–0.831)	0.601 (0.527–0.671)	0.759 (0.720–0.796)	0.645 (0.586–0.699)	0.697 (0.623–0.770)	0.787 (0.743–0.829)
MLP	0.798 (0.754–0.842)	0.631 (0.561–0.702)	0.763 (0.726–0.800)	0.660 (0.602–0.716)	0.693 (0.619–0.760)	0.798 (0.753–0.841)

RF, random forest; DT, decision tree; NN, neural network; NB, naive bayes.

### Interpretability analysis

#### SHAP analysis

**FIGURE 4:**
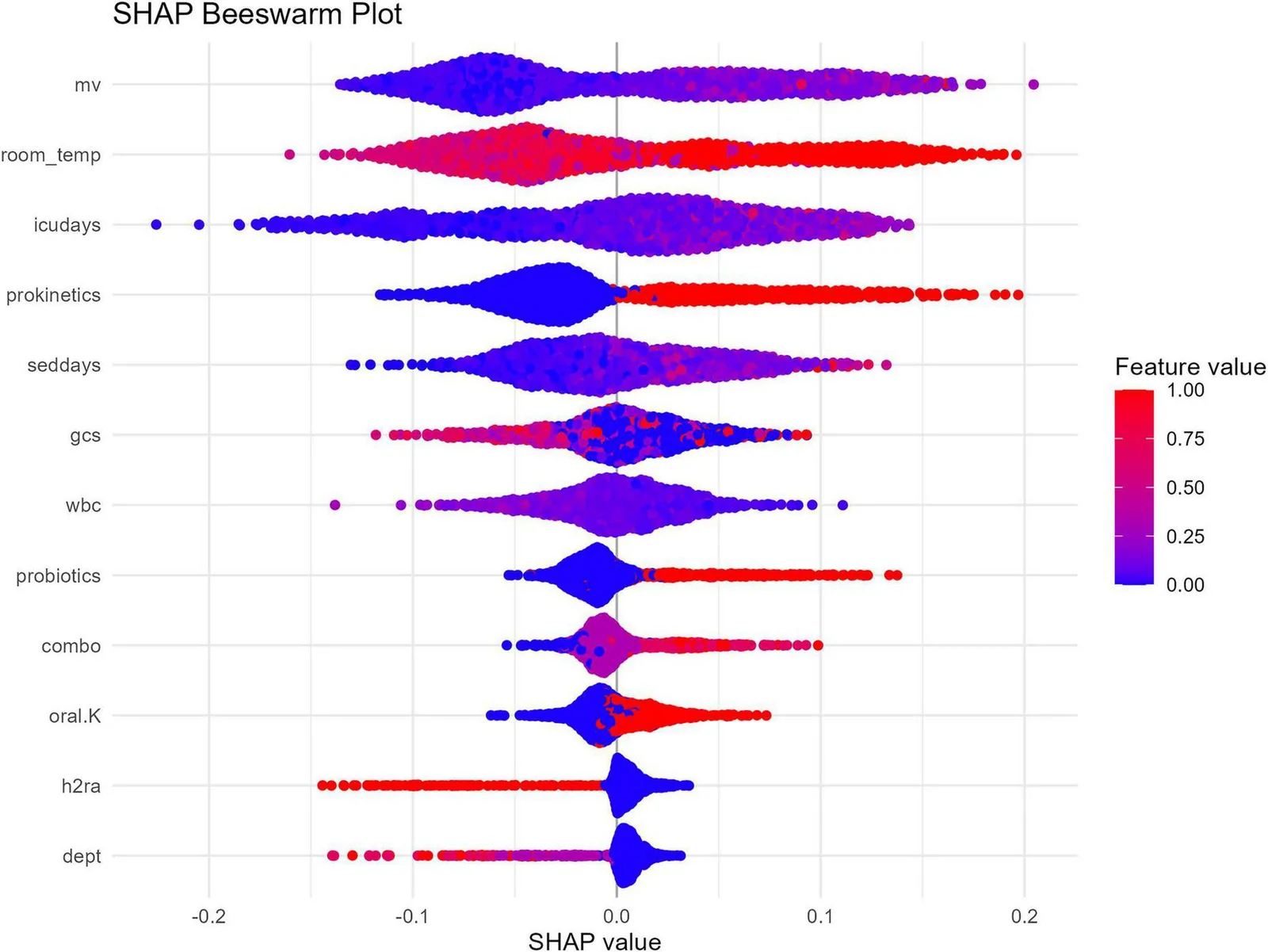
SHapley Additive exPlanations (SHAP) beeswarm plot showing the 12 most important features of the Random Forest model for predicting enteral nutrition-associated diarrhea (ENAD) risk in intensive care unit (ICU) patients.

The SHAP beeswarm plot showed that mechanical ventilation duration, room temperature, and ICU length of stay were the most important contributors to ENAD prediction, followed by prokinetic agent use and sedation duration (Figure 4).

**FIGURE 5:**
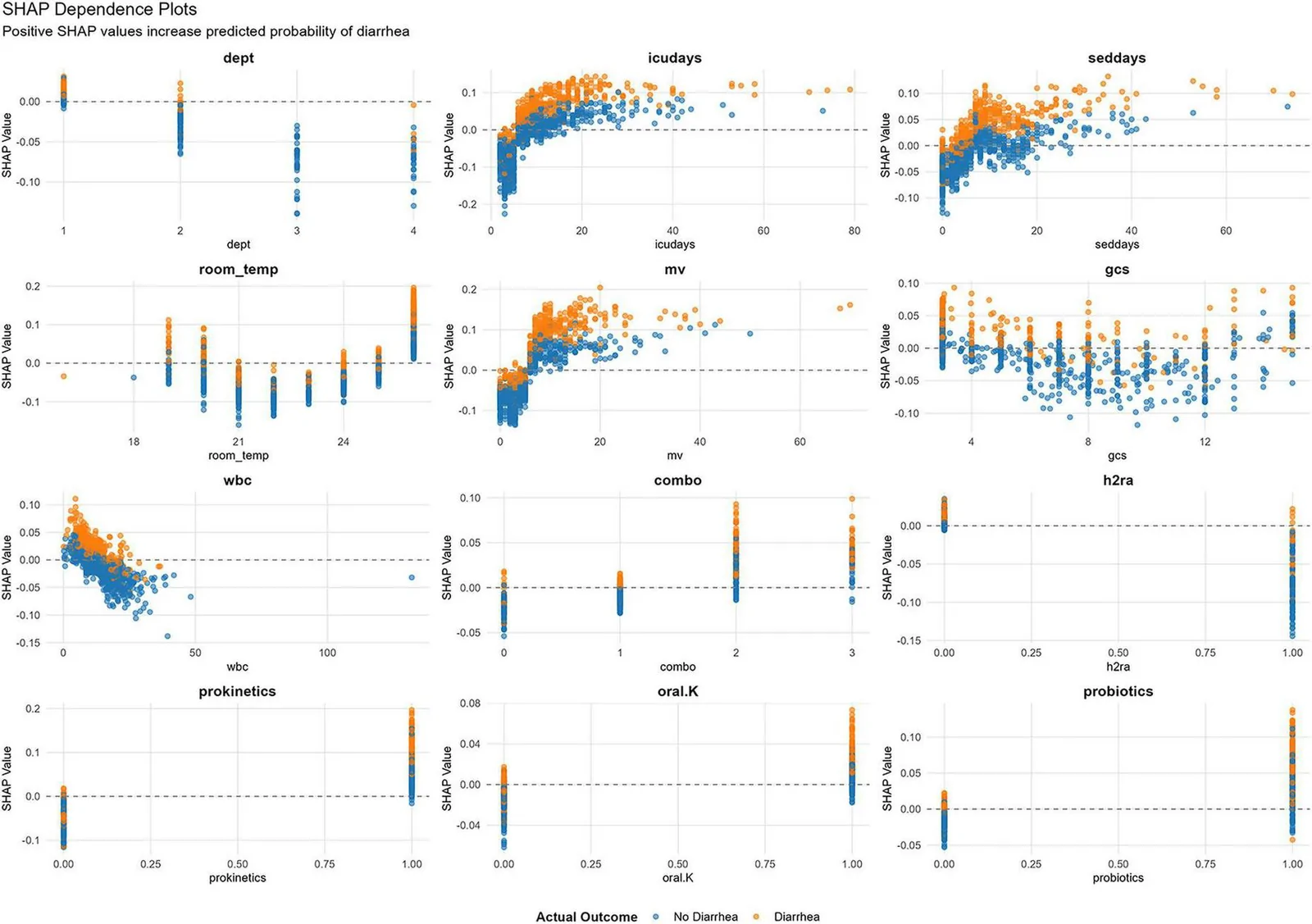
SHapley Additive exPlanations (SHAP) dependence plots showing the effects of features on model predictions for individual patients.

SHapley Additive exPlanations dependence plots showed that longer ICU stay, prolonged mechanical ventilation, longer sedation duration, and higher room temperature were generally associated with increased predicted ENAD risk (Figure 5). Prokinetic agents, probiotics, and H_2_-receptor antagonists also contributed positively to ENAD prediction, whereas WBC showed an overall negative association with predicted risk. GCS score, potassium supplementation route, combined antibiotic therapy, and ICU admission unit showed heterogeneous effects without clear monotonic trends.

The SHAP force plot illustrated individual-level feature contributions (Supplementary Figure 1). In a representative high-risk case, the predicted ENAD probability was 0.962, mainly driven by prolonged mechanical ventilation, higher room temperature, prokinetic agent use, and longer ICU stay.

#### Calibration curve

**FIGURE 6:**
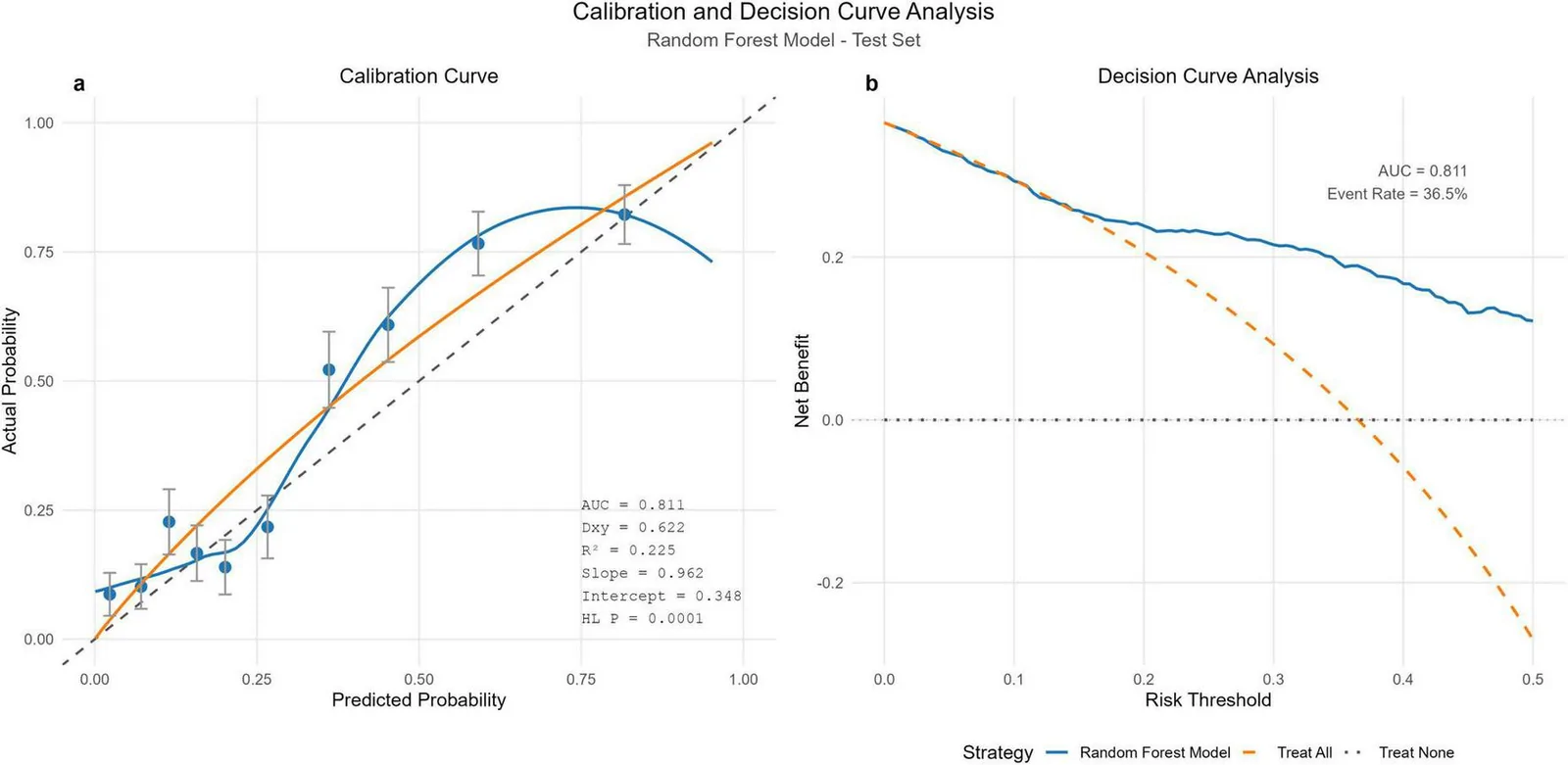
Calibration curve and decision curve analysis of the Random Forest model for predicting enteral nutrition-associated diarrhea (ENAD) in intensive care unit (ICU) patients. **(a)** Calibration curve of the Random Forest model in the test set. **(b)** Decision curve analysis (DCA) of the Random Forest model.

In the test set, the calibration curve showed acceptable agreement between the RF model-predicted ENAD risk and observed outcomes (Figure 6a). The calibration slope was 0.962, close to the ideal value of 1, while the intercept was 0.348, indicating mild systematic calibration bias. Although the Hosmer–Lemeshow test was significant (*p* = 0.0001), this result may reflect its sensitivity to sample size. Overall, the model demonstrated acceptable calibration, and similar calibration trends in the training and test sets suggested no obvious overfitting (Supplementary Figure 2).

#### Decision curve analysis

Decision curve analysis showed that the RF model provided clinical net benefit across a broad range of threshold probabilities in the test set, particularly between 0.10 and 0.50 (Figure 6b). Within this range, the model outperformed both the “treat-all” and “treat-none” strategies, indicating potential value for identifying patients at high risk of ENAD. Similar DCA trends were observed in the training and test sets, although the net benefit was lower in the test set, suggesting the need for further external validation (Supplementary Figure 3).

#### External validation result

**FIGURE 7:**
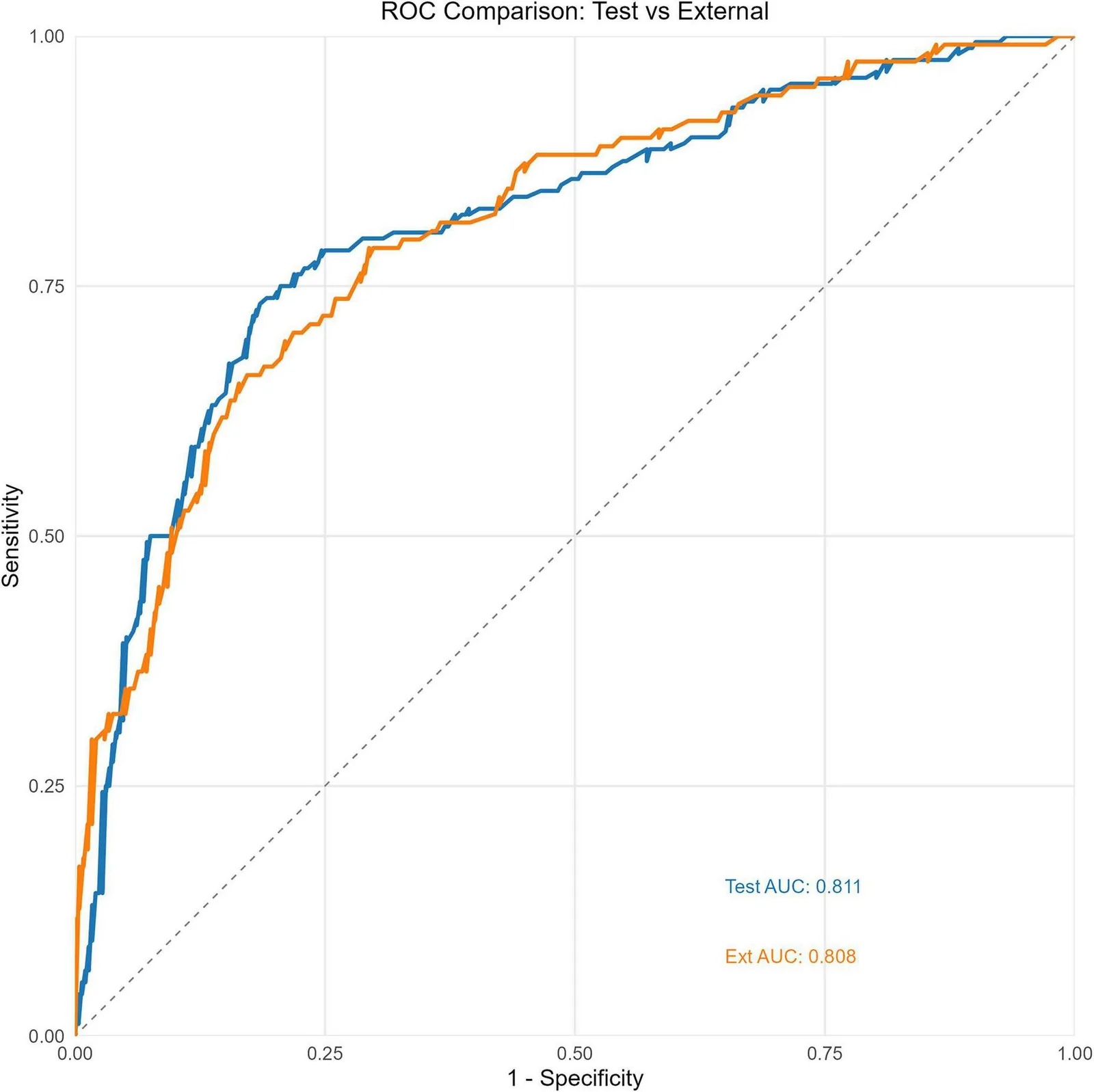
Receiver operating characteristic (ROC) curves comparing model performance in the test set and external validation set.

The external validation cohort included 356 patients, of whom 118 developed ENAD, with an event rate of 33.1% (Table 2). The model achieved an AUC of 0.808 (95% CI: 0.759–0.856), comparable to that in the test set, indicating good external stability. Recall and NPV were 0.780 (95% CI: 0.700–0.851) and 0.866 (95% CI: 0.818–0.912), respectively, suggesting stable performance in identifying ENAD cases and excluding low-risk patients. Accuracy, precision, and F1 score were 0.730, 0.568, and 0.657, respectively, indicating some reduction in positive predictive performance in the external cohort. The ROC curves of the test and external validation sets showed similar trends with substantial overlap (Figure 7), supporting acceptable generalizability. The calibration curve was close to the ideal line, with a calibration slope of 1.046 and a non-significant Hosmer–Lemeshow test (*p* = 0.2253), indicating acceptable calibration (Supplementary Figure 4a). DCA showed positive net benefit within a threshold range of approximately 0.10–0.50, suggesting potential clinical utility (Supplementary Figure 4b).

**TABLE 2:** Model performance metrics in the test set and external validation set.

Dataset	AUC (95% CI)	Recall (95% CI)	Accuracy (95% CI)	F1 score (95% CI)	Precision (95% CI)	NPV (95% CI)
	0.811 (0.769–0.853)	0.786 (0.722–0.842)	0.765 (0.724–0.802)	0.710 (0.661–0.759)	0.647 (0.585–0.713)	0.859 (0.813–0.900)
	0.808 (0.759–0.856)	0.780 (0.700–0.851)	0.730 (0.685–0.772)	0.657 (0.591–0.714)	0.568 (0.487–0.639)	0.866 (0.818–0.912)

## Discussion

To improve the interpretability of the random forest model, SHAP analysis was used to quantify the contribution of each predictor to the model output. SHAP provides both global- and individual-level explanations of model predictions and may help clinicians understand how specific variables contribute to the model-estimated risk of ENAD (30). However, SHAP values reflect associations within the fitted model rather than causal effects. When predictors are correlated, their contributions may also be shared or redistributed. Therefore, SHAP findings should be interpreted in conjunction with the clinical context, correlations among predictors, and the underlying data structure.

SHapley Additive exPlanations analysis showed that the duration of mechanical ventilation, room temperature, and ICU length of stay were the most important predictors of ENAD in ICU patients receiving enteral nutrition. These variables may constitute a clinically meaningful set of indicators for ENAD risk assessment; however, their predictive importance should be interpreted as model-derived associations rather than direct causal effects (31). Prolonged mechanical ventilation may reflect more severe systemic inflammation, metabolic stress, hemodynamic instability, immobilization, and increased exposure to sedatives, vasoactive agents, or antibiotics (32). These conditions may be accompanied by gastrointestinal dysmotility, impaired digestion and absorption, disruption of the intestinal mucosal barrier, and reduced tolerance to enteral nutrition, which may partly explain their association with a higher model-estimated probability of ENAD (33). These conditions may be accompanied by gastrointestinal dysmotility, impaired intestinal barrier function, and reduced tolerance to enteral nutrition. Room temperature was identified as an environment-related model predictor. Higher ambient temperatures may facilitate microbial growth in enteral nutrition formulas under certain feeding conditions; however, feeding-system characteristics, formula hang time, and microbial contamination were not directly assessed. The proposed mechanism therefore remains speculative (16). ICU length of stay may serve as a surrogate marker of disease severity, treatment complexity, and cumulative exposure to ICU interventions. Reverse causality cannot be excluded because ENAD itself may prolong ICU hospitalization (10). More generally, the interpretation of duration-related predictors depends on whether they were measured within the predefined predictor ascertainment window.

Variables related to treatment and medication use also contributed to ENAD prediction. Prokinetic agent use may reflect both altered gastrointestinal motility and pre-existing feeding intolerance, and may therefore be subject to confounding by indication (34, 35). A longer duration of enteral nutrition may represent greater cumulative exposure to formula- and infusion-related factors, but may also reflect prolonged illness or reverse causality (36). Similarly, the contributions of sedation duration, combined antibiotic therapy, oral potassium supplementation, and H_2_-receptor antagonist use to model predictions may be influenced by pharmacological effects, disease severity, treatment indications, and other clinical factors (37). Probiotic use was not consistently associated with a lower model-estimated probability of ENAD, which may be related to heterogeneity in probiotic strains, dosage, timing of administration, antibiotic exposure, and patient characteristics (38). Because this was an observational prediction study, these findings should not be interpreted as evidence of causal or preventive effects of the corresponding treatments.

Department, GCS score, and white blood cell count also contributed to model predictions. Department may represent differences in patient case mix, treatment practices, and nursing procedures rather than an independent biological determinant of ENAD. The contribution of GCS score to model output varied across patients and should be interpreted in conjunction with neurological status, mechanical ventilation, medication exposure, and feeding tolerance (39, 40). White blood cell count may partly reflect systemic inflammatory or stress responses, but it may also be affected by infection, medication use, and other clinical factors. Therefore, its contribution should be regarded as non-specific predictive information rather than evidence of a direct inflammatory mechanism (41).

Notably, although demographic characteristics, comorbidities, ICU admission diagnoses, and disease severity indicators were included as candidate predictors, most were not retained after LASSO selection. This does not imply that these factors are clinically unrelated to ENAD. Rather, their predictive information may overlap with variables more directly reflecting current disease burden and treatment exposure. When predictors are correlated, LASSO tends to retain those with stronger incremental predictive value while shrinking the coefficients of related variables toward zero (42, 43). Because feature selection is influenced by cohort characteristics, variable coding, and the regularization parameter, these findings should be interpreted as cohort-specific predictive patterns rather than evidence of absent biological or clinical associations (44).

Regarding calibration, the Hosmer–Lemeshow test was statistically significant in the internal test set (*p* = 0.0001). However, because this test is sensitive to sample size, the result alone should not be interpreted as evidence of severe miscalibration (45, 46). The calibration slope was close to 1 (0.962), whereas the intercept of 0.348 suggested possible systematic bias in risk estimation. In the external validation set, the calibration slope was 1.046, and the Hosmer–Lemeshow test was not statistically significant (*p* = 0.2253), indicating no clear evidence of marked miscalibration. Overall, the model showed generally acceptable calibration, although further recalibration may be required before clinical implementation.

In the present study, a random forest model combined with SHAP analysis was used to preliminarily identify important variables associated with ENAD prediction and to assess their relative contributions to the model output. By integrating routinely available clinical information from the ICU, the model may support early risk stratification for ENAD and provide supplementary information for subsequent risk assessment and individualized nutritional management (47). Compared with assessment approaches that rely primarily on clinical experience, the model enables the quantitative integration of multidimensional clinical information and may thereby improve the consistency and transparency of risk assessment to some extent (48). The model does not require additional specialized examinations. However, this retrospective study did not evaluate its performance within actual clinical workflows or its effects on clinical decision-making and patient outcomes. Therefore, the available evidence remains insufficient to support routine clinical implementation. Further prospective implementation studies are required to validate its clinical applicability.

## Limitations

This study has several limitations. First, although a multicenter design and independent external validation were used, the retrospective observational design may have introduced incomplete documentation, information bias, selection bias, and residual confounding. Therefore, prospective validation is still required.

Second, some potentially relevant ENAD-related variables, such as formula warming, feeding infusion rate, antibiotic type, and antibiotic duration, were not included, which may have limited the comprehensiveness and predictive performance of the model.

Third, the current model was based primarily on static clinical variables. For repeatedly measured laboratory indicators, only the eligible value closest to EN initiation and recorded before ENAD onset was included. Although this approach standardized the temporal reference point, it may have omitted clinically relevant longitudinal information, such as trends, variability, and transient abnormalities. Therefore, the model may support static early risk stratification but cannot replace dynamic real-time prediction. Future prospective studies should incorporate longitudinal clinical data and dynamic prediction methods.

Fourth, although the calibration curves suggested generally acceptable agreement between predicted and observed risks, the significant Hosmer–Lemeshow test in the internal test set and the calibration intercept indicated possible residual miscalibration and systematic prediction bias. Recalibration in larger and more diverse populations may therefore be required before clinical implementation.

Finally, although SHAP enhanced model interpretability by quantifying the contribution of individual predictors to model outputs, it may not fully characterize complex non-linear interactions or synergistic effects among correlated variables. Future studies should incorporate feature interaction analyses and multimodal or longitudinal data to further improve model interpretability and clinical applicability.

## Conclusion

This study developed and evaluated machine learning models for predicting ENAD risk in ICU patients receiving enteral nutrition. The incidence of ENAD was 34.8%. LASSO regression selected 12 features from the initial 42 candidate predictors, encompassing clinical status, therapeutic interventions, medication use, environmental factors, and laboratory indicators. Among the evaluated models, the random forest model based on these 12 features showed the best performance, with AUCs of 0.811 (95% CI, 0.769–0.853) in the internal test set and 0.808 (95% CI, 0.759–0.856) in the external validation cohort, indicating acceptable discrimination and relatively stable performance across cohorts. The model also demonstrated acceptable calibration and potential clinical utility. SHAP analysis identified the variables contributing most strongly to model predictions and enhanced model interpretability. These findings suggest that the model may support static early risk stratification and inform targeted nutritional care for critically ill patients receiving enteral nutrition.

## Data Availability

The data analyzed in this study is subject to the following licenses/restrictions: the raw data are not publicly available due to privacy and ethical restrictions. Requests for relevant data may be directed to the corresponding author, subject to institutional and ethical approval. Requests to access these datasets should be directed to Xia⋅Li, jzlx6565@163.com.

## Supplementary material

Supplementary Figure 1 Example of a single-sample SHAP force plot.

Supplementary Figure 2 Calibration curves of the random forest model in the training and testing datasets.

Supplementary Figure 3 Decision curve analysis of the random forest model in the training and testing datasets.

Supplementary Figure 4 Calibration curve and decision curve analysis of the random forest model in the external validation set.
