# Supplementary material for: Development and validation of an interpretable machine learning model for predicting enteral nutrition-associated diarrhea in ICU patients: a multicenter study
Source: Front Nutr. 2026 Aug 17;13:1896922. doi: 10.3389/fnut.2026.1896922 (PMC13526544; doi:10.3389/fnut.2026.1896922)
Supplement: Supplementary file 1 [file Supplementary_file_1.pdf]

Random Forest (RF):

Random Forest hyperparameter tuning was performed using 10-fold cross-validation (10-fold CV) with grid search. The main hyperparameter optimized was mtry, which defines the number of variables randomly sampled at each split. The search range for mtry was set as 2, 3, 4, 5, and 6, based on the total number of features (12 features). Model performance was evaluated using the ROC-AUC metric. The optimal hyperparameter combination selected by the highest cross-validation ROC-AUC was:

mtry = 2

ntree = 500

nodesize = 5

Decision Tree (DT):

Decision Tree (DT) hyperparameter tuning was conducted using 10-fold cross-validation (10-fold CV) with grid search. The complexity parameter (cp) was optimized to control tree pruning and avoid overfitting. The search range for cp was set from 0.001 to 0.1 with a step size of 0.002. The splitting criterion was **information gain**. Model performance was evaluated using the ROC-AUC metric. The optimal parameter selected by the highest cross-validation ROC-AUC was:

cp = 0.005

Support Vector Machine (SVM):

Support Vector Machine (SVM) with a radial basis function (RBF) kernel was implemented. Hyperparameter tuning was performed using 10-fold cross-validation (10-fold CV) with grid search. The optimized hyperparameters were the penalty

parameter C and the kernel coefficient sigma. The search ranges were set as C = 0.1, 1, 5, 10, 20 and sigma = 0.001, 0.01, 0.1, 1. Model performance was evaluated using the ROC-AUC metric. The optimal hyperparameters selected by the highest cross-validation ROC-AUC were:

C = 5

sigma = 0.01

Extreme Gradient Boosting (XGBoost):

For the eXtreme Gradient Boosting (XGBoost) model, hyperparameter tuning was conducted using 10-fold cross-validation (10-fold CV) with grid search. The tuning parameters included max_depth, eta, and gamma. The search ranges were set as:

max_depth = 3, 5, 7

eta = 0.05, 0.1, 0.2

gamma = 0, 0.1

Adaptive Boosting (AdaBoost):

Adaptive Boosting (AdaBoost) classification models were established using decision stumps (maxdepth = 1). Hyperparameter tuning was performed using 10-fold cross-validation (10-fold CV) with grid search; the optimized parameters included the number of iterations (iter) and the learning rate (nu), with search ranges set as iter = 50, 100, 150, 200 and nu = 0.01, 0.1, 0.5, 1, while the maximum tree depth was fixed at 1. Model performance was evaluated using the ROC-AUC metric, and the optimal hyperparameters identified by the highest cross-validation AUC were:

iter = 150

nu = 0.50

maxdepth = 1

K-Nearest Neighbors (KNN):

k-Nearest Neighbors (k-NN) classification models were established. Hyperparameter tuning was performed using 10-fold cross-validation (10-fold CV) with grid search. The optimized parameter was the number of nearest neighbors (k). The search range was set as k = 1, 3, 5, 7, 9, 11, 13, 15, 17, 19, 21. Model performance was evaluated using the ROC-AUC metric, and the optimal hyperparameter identified by the highest cross-validation AUC was:

k = 21.

LightGBM:

Light Gradient Boosting Machine (LightGBM) hyperparameter tuning was performed using 10-fold cross-validation (10-fold CV) with grid search. The main hyperparameters optimized were num_leaves, learning_rate, feature_fraction, bagging_fraction, min_data_in_leaf, and max_depth. The search ranges were set as num_leaves = 31, 63; learning_rate = 0.01, 0.05, 0.1; feature_fraction = 0.8, 1.0; bagging_fraction = 0.8, 1.0; min_data_in_leaf = 20, 30; max_depth = -1, 5, based on the total number of features (12 features). Model performance was evaluated using the ROC-AUC metric. The optimal hyperparameter combination selected by the highest cross-validation ROC-AUC was:

num_leaves = 31

learning_rate = 0.05

feature_fraction = 0.8

bagging_fraction = 1.0

min_data_in_leaf = 20

ax_depth = -1

nrounds = 100

Neural Network：

Neural Network (NN) hyperparameter tuning was performed using 10-fold cross-validation (10-fold CV) with grid search. The main hyperparameters optimized were size (number of hidden units) and decay (weight decay regularization parameter). The search range for size was set as 1, 3, 5, 7, 9 and decay was set as 0, 0.001, 0.01, 0.1, based on the total number of features (12 features). Model performance was evaluated using the ROC-AUC metric. The optimal hyperparameter combination selected by the highest cross-validation ROC-AUC was:

size = 3

decay = 0.1

Naive Bayes :

Naive Bayes (NB) hyperparameter tuning was performed using 10-fold cross-validation (10-fold CV) with grid search. The main hyperparameter optimized was laplace, which defines the Laplace smoothing parameter. The search range for laplace was set as 0, 0.5, 1, 2, and 5, based on the total number of features (12 features). The parameters usekernel and adjust were fixed at FALSE and 1, respectively, to maintain the standard naive Bayes form. Model performance was evaluated using the ROC-AUC

metric. The optimal hyperparameter combination selected by the highest cross-validation ROC-AUC was:

laplace = 0.0

usekernel = FALSE

adjust = 1

ANN:

Artificial Neural Network (ANN) hyperparameter tuning was performed using 10-fold cross-validation (10-fold CV) with grid search. The main hyperparameters optimized were size (number of hidden units) and decay (weight decay regularization parameter). The search range for size was set as 3, 5, 7, 9 and decay was set as 0, 0.01, 0.05, 0.1, based on the total number of features (12 features). The maximum number of iterations (maxit) was fixed at 1000 for model convergence. Model performance was evaluated using the ROC-AUC metric. The optimal hyperparameter combination selected by the highest cross-validation ROC-AUC was:

size = 5

decay = 0.1

maxit = 1000

MLP:

Multilayer Perceptron (MLP) hyperparameter tuning was performed using 10-fold cross-validation (10-fold CV) with grid search. The main hyperparameters optimized were size (number of hidden units) and decay (weight decay regularization parameter). The search range for size was set as 1, 2, 3 and decay was set as 0, 0.001, 0.01, 0.1,

based on the total number of features (12 features). The parameters maxit and MaxNWts were fixed at 100 and 10000, respectively. Model performance was evaluated using the ROC-AUC metric. The optimal hyperparameter combination selected by the highest cross-validation ROC-AUC was:

size = 2

decay = 0.1

maxit = 100

MaxNWts = 10000
