# Supplementary material for: Development and validation of an interpretable machine learning model for predicting enteral nutrition-associated diarrhea in ICU patients: a multicenter study
Source: Front Nutr. 2026 Aug 17;13:1896922. doi: 10.3389/fnut.2026.1896922 (PMC13526544; doi:10.3389/fnut.2026.1896922)
Supplement: Supplementary file 2 [file Table_1.docx]

Table S1. Demographic and clinical characteristics of critically ill ICU patients with and without enteral nutrition-associated diarrhea.

| Characteristics |  | Subgroups | All patients  (n=1536) | Non-ENAD  (n=1002) | ENAD  (n=534) | P value |
| --- | --- | --- | --- | --- | --- | --- |
| Gender |  | Meal | 1019(66.3) | 664(66.3) | 355(66.5) | 0.933 |
|  |  | Female | 517(33.7) | 338(33.7) | 179(33.5) |  |
| Marital status |  | Single | 50(3.2) | 31(3.1) | 19(3.6) | 0.670 |
|  |  | Married | 1391(90.6) | 913(91.1) | 478(89.5) |  |
|  |  | Divorced | 37(2.4) | 21(2.1) | 16(3.0） |  |
|  |  | Widowed | 58(3.8) | 37(3.7) | 21(3.9) |  |
| Admission type |  | Emergency | 1142(74.30 | 780(77.8) | 362(67.8) | <0.001 |
|  |  | Outpatient | 25(1.7) | 13(1.3) | 12(2.2) |  |
|  |  | Transfer | 369(24) | 209(20.9) | 160(30.0） |  |
| Department |  | ICU | 1315(88.0) | 847(84.5） | 504(94.4) | <0.001 |
|  |  | NICU | 104(6.8) | 82(8.20 | 22(4.10 |  |
|  |  | EICU | 47(3.1) | 44(4.4) | 3(0.6) |  |
|  |  | RICU | 34(2.1） | 29(2.9) | 5(0.9) |  |
| Diagnosis |  | Neuro | 776(50.5) | 486(48.5) | 290(54.3） | 0.259 |
|  |  | Circulatory | 122(7.9) | 89(8.8) | 33(6.1) |  |
|  |  | Respiratory | 295(19.2) | 193(19.3) | 102(19.1) |  |
|  |  | Infectious | 32(2.1) | 22(2.2) | 10(1.9） |  |
|  |  | Metabolic | 27(1.8) | 17(1.7） | 10(2.9） |  |
|  |  | Urinary | 36(2.3) | 22(2.2) | 14(2.6） |  |
|  |  | Digestive | 99(6.4) | 72(7.2) | 27(5.1） |  |
|  |  | Others | 149(9.8） | 101(10.1) | 48(9.0） |  |
| GI_hx |  | NO | 1236(80.5) | 783(78.1) | 453(84.8) | 0.002 |
|  |  | YES | 300(19.5) | 219(21.9) | 81(15.3) |  |
| DM_hx |  | NO | 1228(79.9) | 805(80.3) | 423(79.2） | 0.600 |
|  |  | YES | 308(20.1) | 197(19.7) | 111(20.8） |  |
| HTN_hx |  | NO | 765(49.8) | 510(50.9) | 255(47.8) | 0.240 |
|  |  | YES | 771(50.2) | 492(49.1) | 279(52.2) |  |
| FA_hx |  | NO | 1524(99.2) | 992(99.0) | 532(99.6) | 0.309 |
|  |  | YES | 12(0.8) | 10(1.0) | 2(0.4) |  |
| CA_hx |  | NO | 1484(96.6) | 964(96.2) | 520(97.4) | 0.227 |
|  |  | YES | 52(3.4) | 38(3.8) | 14(2.6) |  |
| NRS2002 |  | Negative | 239(15.6) | 170(17.0) | 69(12.9) | 0.037 |
|  |  | Positive | 1297(84.4) | 832(83.0) | 465(87.1) |  |
| FOBT |  | Negative | 1514(98.6) | 994(99.2) | 520(97.4) | 0.006 |
|  |  | Positive | 21(1.3) | 7(0.7） | 14(2.6) |  |
|  |  | Gross Blood | 1(0.1) | 1(0.1) | 0 |  |
| EN type |  | TPF | 924(60.2) | 608(60.7) | 316(59.2) | <0.001 |
|  |  | SP | 251(16.3) | 201(20.1) | 50(9.4) |  |
|  |  | TP-HE | 182(11.8) | 93(9.3) | 89(16.7) |  |
|  |  | Mixed feeding | 179(11.7) | 100(9.9) | 79(14.7) |  |
| EN admin method |  | Continuous | 1431(93.2） | 932(93.0) | 499(93.4) | 0.056 |
|  |  | Intermittent | 41(2.7) | 33(3.3) | 8(1.5) |  |
|  |  | Bolus | 64(4.1) | 37(3.7) | 27(5.1) |  |
| Tube type |  | NGT | 1384(90.1) | 901(89.9) | 483(90.4) | 0.573 |
|  |  | NJT | 150(9.8) | 99(9.9) | 51(9.6) |  |
|  |  | G-tube | 2(0.1) | 2(0.2) | 0 |  |

Table S1. Continued

| Characteristics |  | Subgroups | All patients  (n=1536) | Non-ENAD  (n=1002) | ENAD  (n=534) | P value |
| --- | --- | --- | --- | --- | --- | --- |
| Abx |  | NO | 93(6.1) | 78(7.8) | 15(2.8) | <0.001 |
|  |  | YES | 1443(93.9) | 924(92.2) | 519(97.2) |  |
| Abx route |  | IV | 1410(91.8) | 893(89.1) | 517(96.8) | <0.001 |
|  |  | PO | 1(0.1) | 0 | 1(0.2) |  |
|  |  | EN | 32(2.0) | 31(3.1) | 1(0.2) |  |
|  |  | NO | 93(6.1） | 78(7.8) | 15(2.8) |  |
| Abx combo |  | NO | 131(8.5) | 105(10.5) | 26(4.9) | <0.001 |
|  |  | Single | 1073(69.9) | 735(73.4) | 338(63.3) |  |
|  |  | Dual | 233(15.2) | 121(12.1) | 112(21.0) |  |
|  |  | Triple+ | 99(6.4) | 41(4.0) | 58(10.8) |  |
| RRT |  | NO | 1286(83.7) | 854(85.2) | 432(80.9) | 0.029 |
|  |  | YES | 250(16.3) | 148(14.8) | 102(19.1) |  |
| PPI |  | NO | 556(36.2) | 355(35.4) | 201(37.6) | 0.390 |
|  |  | YES | 980(63.8) | 647(64.6) | 333(62.4) |  |
| H2RA |  | NO | 1407(91.6) | 902(90.0) | 505(94.6) | 0.002 |
|  |  | YES | 129(8.4) | 100(10.0) | 29(5.4) |  |
| Prokinetics |  | NO | 984(64.1) | 723(72.2) | 261(48.9) | <0.001 |
|  |  | YES | 552(35.9) | 279(27.8) | 273(51.1) |  |
| Oral potassium |  | NO | 926(60.3) | 653(65.2) | 273(51.1) | <0.001 |
|  |  | YES | 610(39.7) | 349(34.8) | 261(48.9) |  |
| Probiotics |  | NO | 1209(78.7) | 821(81.9) | 388(72.7) | <0.001 |
|  |  | YES | 327(21.3) | 181(18.1) | 146(27.3) |  |
| Sedatives |  | NO | 236(15.40 | 193(19.3) | 43(8.10 | <0.001 |
|  |  | YES | 1300(84.6) | 809(80.7) | 491(91.9) |  |
| Opioids |  | NO | 740(48.2) | 497(49.6) | 243(45.5) | 0.126 |
|  |  | YES | 796(51.8) | 505(50.4) | 291(54.5) |  |
| Age ,M(IQR) |  |  | 67(56-74) | 67(56-75) | 67(56-74) | 0.729 |
| APACHE II ,M(IQR) |  |  | 23(18-27) | 22(17-27) | 24(19-27) | 0.003 |
| GCS ,M(IQR) |  |  | 4(3-8) | 5(3-8) | 3(3-7) | <0.001 |
| MV ,M(IQR) |  |  | 5(2-9) | 3(2-7） | 8(5-12) | <0.001 |
| RoomTemp ,M(IQR) |  |  | 24(22-26) | 24(22-25) | 25(22-26） | <0.001 |
| ICU days ,M(IQR) |  |  | 8(4-14) | 7(4-11) | 10(7-17) | <0.001 |
| EN days ,M(IQR) |  |  | 7(3-12) | 5(3-10) | 9(6-15) | <0.001 |
| Sedative days ,M(IQR) |  |  | 6(2-11) | 4(2-9) | 8(5-14) | <0.001 |
| WBC(×10⁹/L) ,M(IQR) |  |  | 11.48(8.32-16.27) | 11.85(8.49-16.92) | 10.85(8.02-15.24) | 0.003 |
| Hb(g/L) ,M(IQR) |  |  | 112(91-131) | 114(92-132) | 108.06(90-130) | 0.029 |
| Alb(g/L) ,M(IQR) |  |  | 33.3(29.7-37.4) | 33.4(29.9-37.5) | 33.3(29.3-37.2) | 0.361 |
| BUN(mmol/L) ,M(IQR) |  |  | 7.7(5.24-13.3) | 7.7(5.37-13.09) | 7.87(5.1-13.87） | 0.882 |
| Na(mmol/L) ,M(IQR) |  |  | 140(137-144) | 140(136-143) | 141(137-144.1） | 0.003 |
| K(mmol/L) ,M(IQR) |  |  | 3.8(3.5-4.2) | 3.8(3.5-4.2) | 3.8(3.5-4.3) | 0.797 |
| CRP(mg/L),M(IQR) |  |  | 56.7(16.81-117.27) | 55.81(16.95-120) | 59.7(16.3-109) | 0.574 |
| PCT(ng/mL),M(IQR) |  |  | 0.52(0.10-2.99) | 0.52(0.09-3.23) | 0.55(0.11-2.66) | 0.776 |

P < 0.05 is considered a statistically significant difference.Abbreviations used in this study were as follows: ICU for Intensive Care Unit, ENAD for Enteral Nutrition-Associated Diarrhea, APACHE II for Acute Physiology and Chronic Health Evaluation II with a score range of 0–71, GCS for Glasgow Coma Scale with a score range of 3–15, RRT for Renal Replacement Therapy, EN for Enteral Nutrition, GI_hx for gastrointestinal disease history, DM_hx for diabetes mellitus history, HTN_hx for hypertension history, FA_hx for food allergy history, CA_hx for malignant tumor history, NRS2002 for Nutritional Risk Screening 2002, FOBT for fecal occult blood test, Abx for antibiotics, PPI for proton pump inhibitor, CRP for C-reactive protein, and PCT for procalcitonin. Binary variables (coded as 0 = no and 1 = yes) included gastrointestinal disease history, diabetes mellitus history, hypertension history, food allergy history, malignant tumor history, renal replacement therapy, antibiotic use, H2 receptor antagonist use, prokinetic agent use, oral potassium supplementation, probiotic use, sedative use, opioid use, and the primary outcome of diarrhea.Laboratory parameters involved white blood cell count, hemoglobin, albumin, blood urea nitrogen, serum sodium, serum potassium, C-reactive protein and procalcitonin, and all laboratory results were interpreted according to the standard reference ranges.
